# Subacute Combined Degeneration of the Spine: A Case Study of Delayed Diagnosis in the Emergency Department

**DOI:** 10.7759/cureus.67764

**Published:** 2024-08-25

**Authors:** CJ Ryan, Noah Tolby

**Affiliations:** 1 Emergency Department, Banner - University Medical Center Tucson, Tucson, USA

**Keywords:** anchoring bias, urinary retention, subacute combined degeneration, pernicious anemia, vitamin b12, cyanocobalamin

## Abstract

Subacute combined degeneration of the spine (SCDS) is a well-known disease that classically presents with progressive sensory and motor deficits and characteristic magnetic resonance imaging (MRI) findings, leading to its use as a key diagnostic tool. However, clinical and MRI findings in SCDS may be diverse, and thus, a high index of suspicion should be maintained for this disease, which can cause irreversible neurological damage if left untreated. In this article, we report the case of a 29-year-old female with significant recent life stressors and otherwise unremarkable medical history who presented with progressive weakness of the bilateral lower extremities who previously had unremarkable computed tomography (CT) and MRI completed at an outside hospital for the same symptoms, which had since continued to worsen. Her presentation at our emergency department (ED) prompted urgent evaluation with an MR cord compression study and neurology consultation. This workup resulted in an unremarkable preliminary MR read, and she was without anemia in laboratory studies. Given this, she was ultimately discharged with high suspicion for conversion disorder. After an addendum report from radiology with concern for subacute combined degeneration of the spine, she was called back to the ED where further workup revealed pernicious anemia leading to SCDS. This case highlights the importance of maintaining suspicion and avoiding premature closure in patients with reported neurological deficits.

## Introduction

Subacute combined degeneration of the spine (SCDS) is a known complication of vitamin B12 (cyanocobalamin) deficiency, characterized by demyelination of the dorsal and lateral columns of the spinal cord involving the corticospinal and spinocerebellar tracts. The pathophysiology of this demyelination involves the disruption of myelin synthesis, which is essential for the proper functioning of central and peripheral nerve fibers [1]. Earliest signs of vitamin B12 deficiency include paresthesia and areflexia, which are believed to be related to peripheral nerve damage rather than the spinal cord [2]. If left untreated, this can progress to axonal loss, Wallerian degeneration, and permanent neurological damage [3]. Common initial presenting symptoms of subacute combined degeneration include paresthesias, gait disturbances, and limb weakness [4], all of which are non-specific to the subacute combined degeneration disease process. Therefore, a thorough history and physical examination are critical in patients presenting with these symptoms while including this treatable vitamin deficiency in the differential diagnosis. The demyelinating pattern of SCDS can be visualized as a characteristic abnormal hyperintensity of the posterior spinal cord on T2-weighted imaging, often with resolution of these findings on follow-up imaging after appropriate treatment [2,5]. MRI of the spine may be particularly useful in the emergency setting in patients with progressive neurological symptoms in order to rule out other serious and emergent causes of spinal cord pathology [6].

## Case presentation

A 29-year-old female with no reported past medical history presented to our emergency department (ED) via ambulance with two weeks of progressively decreased strength to the bilateral lower extremities associated with new-onset urinary incontinence. At the onset of her symptoms, she noted bilateral foot numbness and weakness, gradually causing difficulty ambulating as the numbness and weakness progressed proximally up the bilateral lower extremities. At the time of presentation to our ED, she required assistance with a wheelchair for completing her activities of daily living (ADLs) and had fallen forward down the stairs at home earlier in the day after her son pulled on her leg. This caused her to land on her right knee and right lateral chest prompting presentation at the ED. She stated that approximately 10-14 days prior, she was evaluated at an outside hospital that completed a workup including computed tomography (CT) and magnetic resonance imaging (MRI) of the spine that did not demonstrate any abnormalities. She was unable to remember which portion of her spine was imaged and was unable to produce paperwork. She was reportedly discharged with a referral to neurology at that time, although she had not yet been seen. She denied recent illness, fever, seizures, substance use, or chronic neurological conditions. She denied taking any medications regularly. Upon review of our prior records, she had been seen for similar symptoms in the ED the year prior with a similarly negative workup, at that time reporting only decreased sensation without accompanying weakness. She reports this episode self-resolved within three months.

On examination, she was noted to be afebrile, mildly tachycardic to the 100s, and mildly hypertensive at 145/97. She was alert and oriented X4 (to person, place, time, and context) with a Glasgow Coma Scale (GCS) score of 15. She had normal cognition and speech without aphasia. Cranial nerves were intact and symmetric bilaterally. Deep tendon reflexes (DTRs) were 2+ over bilateral brachioradialis and absent over patellar and Achilles tendons. Upper extremity strength was 5/5 bilaterally, and lower extremity strength was 0/5 bilaterally (no voluntary hip flexion/extension, knee flexion/extension, or ankle/toe movements). She noted decreased/absent sensation to light touch from the epigastric region inferiorly.

Laboratory results demonstrated leukocytosis to 11.3 without evidence of anemia. There were otherwise no significant electrolyte abnormalities and negative urinalysis. A bladder scan demonstrated urinary retention with a bladder volume greater than 700 mL. MR cord compression study of the spine was completed, and the patient was evaluated by neurology while pending final read. Their interview with the patient revealed multiple ongoing life stressors leading to concern for the development of a somatization or conversion disorder. They were also able to obtain the outside hospital records from her ED visit 14 days prior for these symptoms and confirmed that at that time, CT and MRI of the spine were without acute pathology. Additionally, a preliminary MR cord compression read had resulted at that time from her current visit and revealed no acute findings within the C-spine, T-spine, or L-spine. Given the above findings, the consulting neurology team recommended no further interventions including deferral of lumbar puncture, believing that the patient may be presenting with a functional neurological symptom disorder. Her pain had improved in the ED with Tylenol and fentanyl, and she was ultimately discharged with return precautions and instructions to follow up with her initial neurology referral.

**Figure 1:**
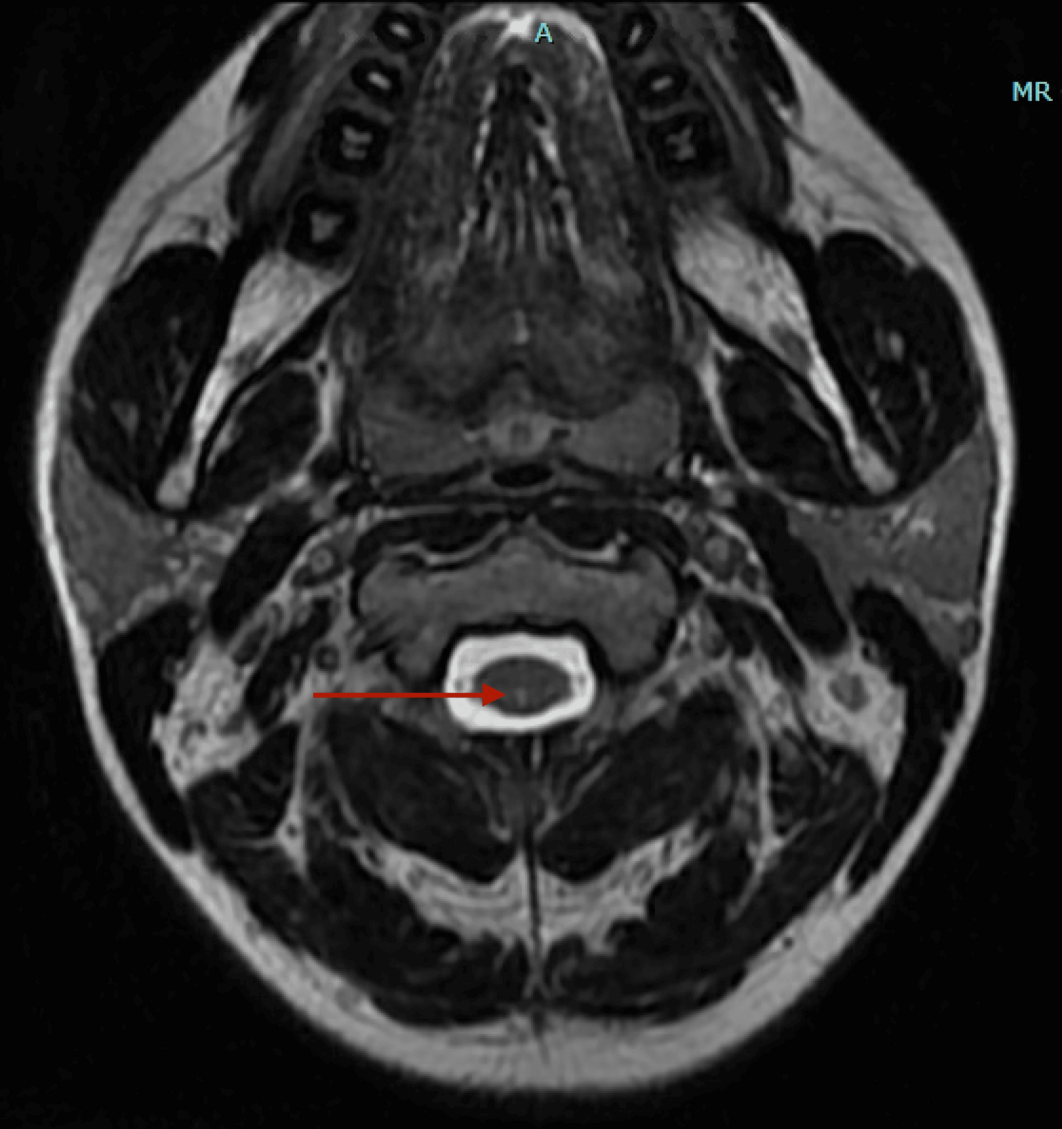
Dedicated MRI of the C-spine demonstrating small subtle short-segment T2 hyperintense signal affecting the dorsal columns C2-C4 concerning for subacute combined degeneration of the cord MRI: magnetic resonance imaging

**Figure 2:**
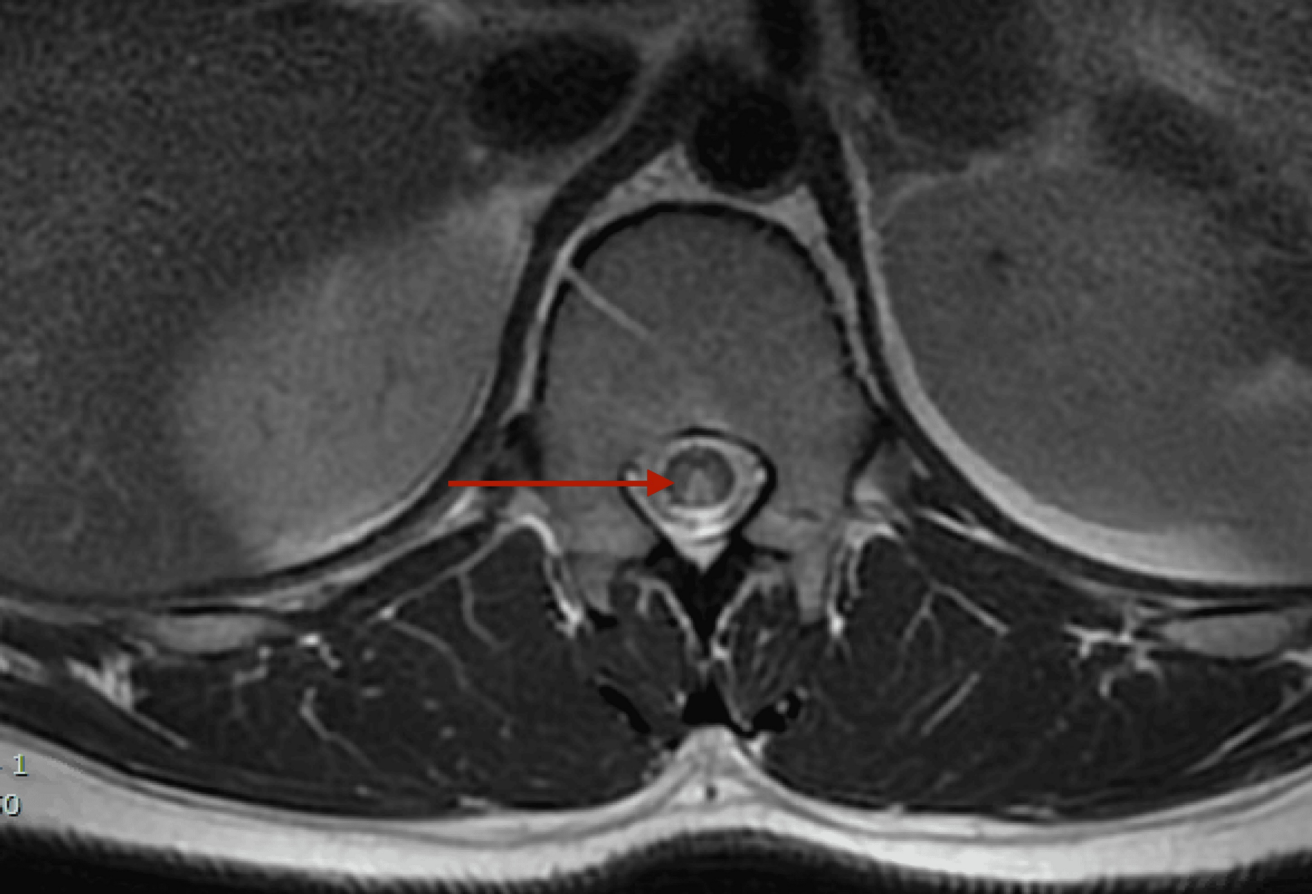
Dedicated MRI of the T-spine demonstrating long-segment subtle T2 hyperintensity within the dorsal aspect of the lower thoracic spinal cord involving the bilateral dorsal columns compatible with the diagnosis of subacute combined degeneration of the cord and may be seen in the setting of underlying metabolic abnormality such as B12 deficiency MRI: magnetic resonance imaging

After her initial discharge from our hospital, a final read with addendum was completed on the MR cord compression study as above, which revealed upon further review diffuse edema throughout the thoracic and lumbar spinal cord, which appeared mildly widened in the anteroposterior (AP) dimension compared to prior study, concerning for acute infectious or inflammatory myeloneuropathy. The patient was contacted via telephone and advised to return to the ED for emergent imaging. She returned the following day for re-evaluation. Repeat laboratory studies revealed resolution of leukocytosis but with new mild anemia with hemoglobin of 11.6 g/dL from 12.5 g/dL with mean corpuscular volume (MCV) stable at a normal high of 100 fL. Follow-up imaging in the ED upon re-evaluation included a dedicated total spine MRI study with and without contrast, which resulted in dorsal column hyperintensity at the C2-C4 and thoracic spine level (Figures 1, 2), compatible with subacute combined degeneration of the spine, secondary to what was at this point suspected B12 deficiency.

The patient was subsequently admitted to neurology service with further workup, revealing a positive intrinsic factor antibody, decreased vitamin B12 level of <150 pg/mL, elevations in methylmalonic acid (10,866) and homocysteine (20.2), and normal folate (15 ng/mL). MRI of the brain was unremarkable and notably negative for parenchymal lesions. These laboratory results in conjunction with characteristic imaging and presentation as above were consistent with a diagnosis of subacute combined degeneration secondary to pernicious anemia. Given her accepted diagnosis of pernicious anemia, she received 1,000 mcg of cyanocobalamin injections daily while inpatient, with a plan for re-evaluation and adjustment of these injections to a weekly and then monthly basis dependent on improvement. She was discharged to a skilled nursing facility based on physical therapy/occupational therapy (PT/OT) recommendations with a Foley catheter in place until able to void without retention. At the time of her ultimate disposition, she had marked improvement in bilateral lower extremity strength and improvement in vibration and pinprick sensation. She was scheduled for close neurology, urology, and primary care follow-up.

## Discussion

Subacute combined degeneration is diagnosed based on clinical presentation and laboratory abnormalities including low vitamin B12 levels or increased levels of metabolites methylmalonic acid and homocysteine. The diagnosis may be aided by characteristic MRI findings including TW2I hyperintensity of the posterior spinal cord [2,5], which typically resolves upon treatment with vitamin B12 repletion [7]. MRI of the spine may be particularly useful in the emergency setting in patients with progressive symptoms to rule out other serious causes of spinal cord pathology. Timely diagnosis of SCD and appropriate treatment with vitamin B12 repletion is necessary to prevent irreversible neurological injury [5,7,8].

In the case of our patient who presented with progressive weakness and paresthesia, she was ultimately prematurely discharged from two emergency departments after unremarkable initial imaging and laboratory studies. Specifically, at our emergency department, she presented with a normal hemoglobin level and unremarkable preliminary read of MR cord compression study, with additional context of normal findings on MRI and CT of the spine from an outside hospital 14 days prior. Of note, MR cord compression is a limited study as compared to a dedicated MR of the spine, which the patient did receive upon return to the ED for further evaluation of suspected SCDS. MR cord compression protocols primarily differ from total spine MRI in that the former utilizes a limited number of sequences targeting the suspected area of compression with fewer planes of view and without contrast enhancement. Thus, the evaluation completed by dedicated MRI of the spine provides a more comprehensive evaluation of spinal pathology through obtaining a more extensive set of contrast and non-contrast sequences. In emergency settings, MR cord compression is often used for the evaluation of suspected spinal cord pathology as it offers the significant advantage of a faster protocol, reducing examination time by 79% while maintaining a high diagnostic yield for cord compression [9]. This is of particular benefit in the emergency department setting as rapid diagnosis is often critical and can significantly change management. Ultimately, the MR cord compression study had proved sufficient in the case of our patient for the detection of spinal cord pathology in the form of hyperintensity in the C2-C4 and thoracic spinal cord levels consistent with SCDS; however, this had been missed on a preliminary read leading to premature disposition. 

In the case of our patient, there was also a component of significant recent life stressors including physical and emotional trauma that she had disclosed to the neurology team who had initially evaluated her in the emergency department. There is a well-documented relationship between trauma and somatization as well as conversion disorders [10,11]. In light of a negative outside hospital spinal imaging, negative preliminary cord compression study from our hospital, and the stated history of recent psychosocial stressors, our patient's pathology was determined to be highly suggestive of a conversion disorder without further interventional recommendations from the consulting neurology service. Indeed, there may have been a component of anchoring bias in this case, as a premature diagnosis was established based on this early memorable piece of information within the initial patient interview [12]. There is evidence in the literature to demonstrate that clinical, electrophysical, and MRI findings in SCDS may be diverse [2], with not all patients exhibiting the characteristic imaging findings discussed above. It is critical to maintain a broad differential diagnosis in the emergency setting for such a presentation including spinal cord compression, cauda equina syndrome, multiple sclerosis, demyelinating polyneuropathy, transverse myelitis, Guillain-Barré syndrome, electrolyte derangements, and vitamin deficiencies. As discussed above and utilized in the case of our patient, a thorough history and physical examination along with imaging and laboratory workup may be necessary to aid in identifying emergent and treatable etiologies. However, as this case illustrates, it is crucial to maintain suspicion for highly treatable conditions such as vitamin B12 deficiency in the differential diagnosis of all peripheral and central neurological disorders including when imaging lacks characteristic features and avoid premature closure prior to collection of all appropriate evidence.

## Conclusions

Subacute combined degeneration is a well-known complication of vitamin B12 deficiency that may present with reversible and non-reversible neurological complications. Here, we present a case of SCDS in the setting of pernicious anemia in a young and otherwise healthy female with significant life stressors, who presented with significant and progressive neurological deficits including lower extremity weakness, numbness, and urinary retention. Despite these significant manifestations, spinal CT and MRI from an outside hospital and a preliminary MR cord compression read from the emergency department were unrevealing, leading to premature discharge without adequate treatment of these presenting symptoms. Maintaining a high index of suspicion in patients with key features of this illness despite unremarkable laboratory and imaging results may help prevent morbidity and mortality.
